# Case Report: Allogenic Simple Limbal Epithelial Transplantation From a Human Leukocyte Antigen-Matched Living Related Donor to Treat Bilateral Corneal Chemical Burns Post Laser-Assisted *in situ* Keratomileusis

**DOI:** 10.3389/fmed.2022.849791

**Published:** 2022-02-25

**Authors:** Yuh-Shin Chang, Tsung-Yueh Chan, Ren-Long Jan, Sung-Huei Tseng

**Affiliations:** ^1^Department of Ophthalmology, Chi Mei Medical Center, Tainan, Taiwan; ^2^Graduate Institute of Medical Science, College of Health Science, Chang Jung Christian University, Tainan, Taiwan; ^3^Department of Ophthalmology, Buddhist Tzu Chi General Hospital, Hualien, Taiwan; ^4^Department of Pediatrics, Chi Mei Medical Center, Liouying, Tainan, Taiwan; ^5^Department of Ophthalmology, National Cheng Kung University Hospital, College of Medicine, National Cheng Kung University, Tainan, Taiwan

**Keywords:** chemical burns, laser-assisted *in situ* keratomileusis, bilateral limbal stem cell deficiency, allogenic simple limbal epithelial transplantation, human leukocyte antigen-matched living related donor, systemic immunosuppressant

## Abstract

A 35-year-old woman who had undergone laser-assisted *in situ* keratomileusis in both eyes experienced bilateral total limbal stem cell deficiency (LSCD) due to chemical burns. Due to bilateral severe LSCD, allogenic simple limbal epithelial transplantation (SLET) from a human leukocyte antigen (HLA)-matched living related donor was the first choice of treatment for her left eye. We report the first case of HLA or ABO matching living related allogenic SLET for permanent restoration of the cornea for bilateral LSCD treatment. Our ABO-HLA-matched living related allogenic SLET alleviation of the systemic immunosuppressant to topical corticosteroids alone. It also came the limitations of prolonged systemic immunosuppressant usage in conjunctival-limbal allografts and keratolimbal allograft.

## Introduction

Human corneal epithelial cells, which are stratified squamous cells that cover the corneal surface, are constantly exposed to the environment and need to be regenerated continuously by stem cells located in the limbus at the border between the cornea and the conjunctiva. Damage to the limbal stem cells can result in limbal stem cell deficiency (LSCD), which is clinically characterized by progressive conjunctivalization, vascularization, persistent epithelial defects, and scarring of the corneal surface and leads to decreased vision or blindness (1–3). The etiologies of LSCD include hereditary or acquired causes such as ocular thermal or chemical burns, trauma, contact lens wear, and autoimmune diseases such as Stevens-Johnson syndrome.

The purposes of the treatment of LSCD are reepithelialization of the corneal surface and improvement of visual acuity. The current options for treatment of LSCD include surgeries using autologous limbal tissue: surgery using a conjunctival-limbal autograft (CLAU), which is a surgical technique that involves harvesting of two conjunctival-limbal biopsy samples and transfer of these samples to the affected eye directly (4), and cultured limbal epithelial transplantation, which is a technique that involves culturing of limbal biopsy samples to produce limbal cell sheets before transplantation (5). It remains a challenge to treat bilateral LSCD. Several alternative surgeries using non-limbal cell types such as the cultured oral mucosal epithelial transplantation (COMET) technique are options for the treatment of bilateral LSCD using autologous cells and these procedures avoid immunosuppression (6). In addition to COMET, techniques using allogenic limbal tissues such as conjunctival-limbal allografts from a living related donor (lr-CLAL) or cadaveric tissue (keratolimbal allograft [KLAL]) have also shown potential in treating bilateral LSCD. However, systemic immunosuppressants are critical for the survival of allograft tissue, and patients should be monitored for adverse systemic effects while taking immunosuppressants in these two procedures (7, 8).

Simple limbal epithelial transplantation (SLET), an innovative technique introduced by Sangwan et al. (9), is a new treatment strategy that harvests a small limbal biopsy sample from the healthy eye and divides the sample into minute explant pieces that are distributed over the human amniotic membrane and glued to the cornea. SLET has the advantages of requiring a smaller biopsy sample, which avoids the risk of developing iatrogenic LSCD in the donor eye in the CLAU procedure, and overcoming the main issues related to the CLET procedure, i.e., the cost and requirement of cell culture laboratories. SLET is quickly gaining attention worldwide and has been described in several indications including primary and recurrent pterygia, LSCD secondary to multiple surgical interventions or ocular surface squamous neoplasia excision, and failed prior limbal stem cell transplantation (10–12). Bilateral LSCD, which occurs owing to severe chemical burns, mucous membrane pemphigoid, and Stevens-Johnson syndrome, makes patients not have any healthy limbal stem cells and makes them unsuitable candidates for treatment with autologous SLET (13). Allogenic SLET, which uses a limbal stem cell source from either cadaveric or living related donors, has also shown potential and may serve as a new option for treating bilateral LSCD. In addition, allogenic SLET may overcome the main issues related to prolonged systemic immunosuppressant usage in the lr-CLAL and KLAL procedures.

To the best of our knowledge, little has been reported on the use of allogenic SLET in the treatment of bilateral LSCD (7, 13–16). Herein, we describe one patient who underwent allogenic SLET from HLA-matched living related donors in both eyes. Of note, this patient with bilateral total LSCD due to chemical burns had undergone laser-assisted *in situ* keratomileusis (LASIK) in both eyes. LASIK is one of the most common keratorefractive surgeries worldwide. The postoperative complications of LASIK include dry eye, residual refractive error, flap striae, flap dislocation, epithelial defects, epithelial ingrowth, corneal ectasia, and different types of keratitis (17). Regarding the association between ocular procedures and dry eye, LASIK is the most common surgery that influences tear film status postoperatively, and post-LASIK dry eye is the most common dry eye after ophthalmic surgeries (18). Severe dry eye can lead to constant insults to the corneal epithelium, severe dysfunction of the precorneal tear film, and persistent ocular surface inflammation (19). Some reports have described successful treatment of severe dry eye-related LSCD with SLET (15). Our study is the first report of allogenic SLET from HLA-matched living-related donors to treat bilateral LSCD after LASIK.

## Case Report

**Figure 1:**
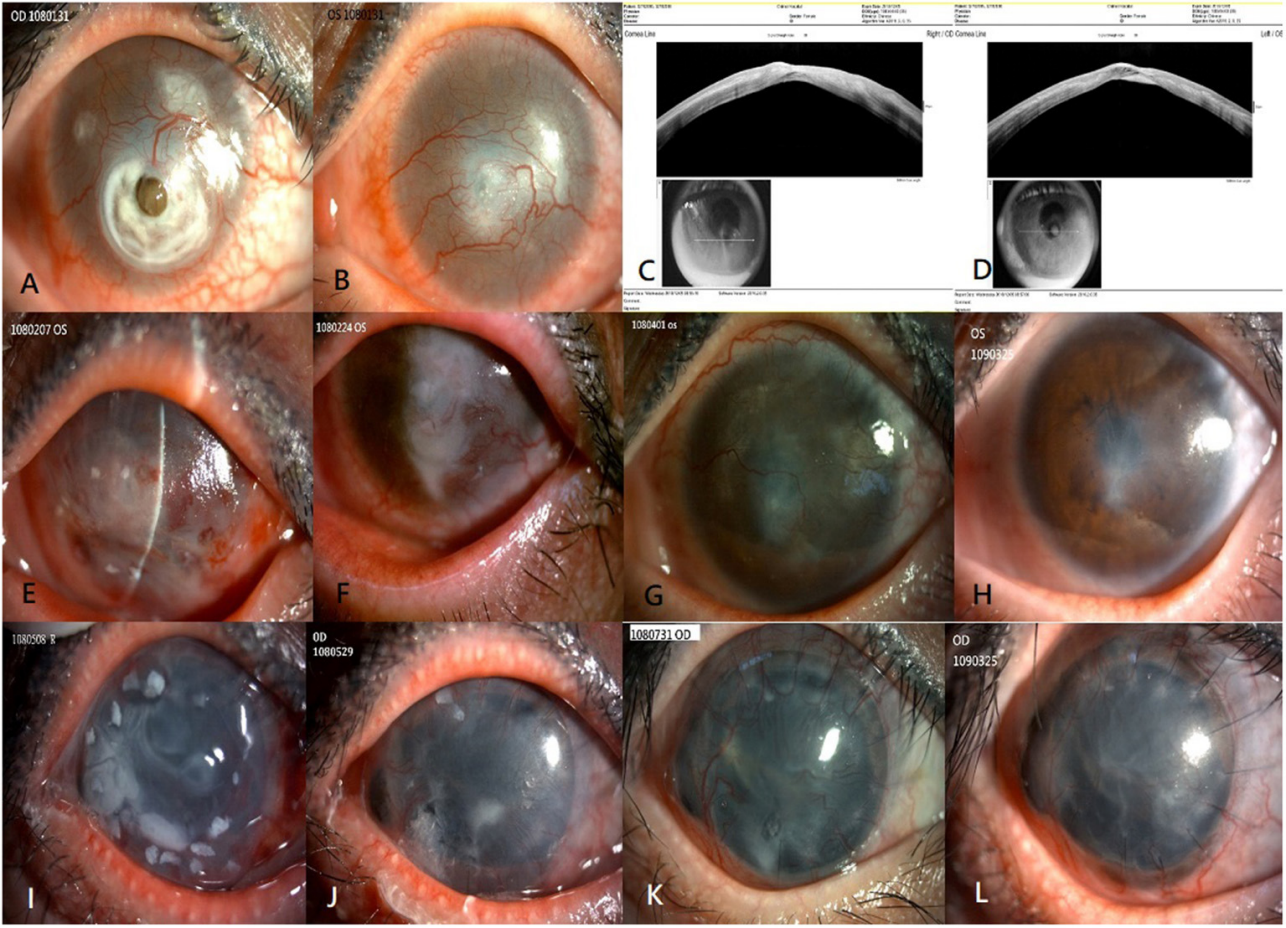
Clinical outcomes of allogenic SLET. The top row shows the preoperative image and anterior segment OCT of both eyes **(A–D)**. Preoperative image showing total LSCD with severely engorged, tortuous neovessel ingrowth onto the cornea, deep stromal scarring of the central cornea in both eyes, and descemetocele formation in the right eye **(A)** and central thinning in the left eye **(B)**. Anterior segment OCT showing flap thinning due to suspicious melting flaps in the right eye **(C)** and left eye **(D)**. The second row from top summarizes the 1-year progressive outcomes post allogenic SLET of the patient's left eye. Postoperative one week image showing circumscribed arranged limbal transplants and amniotic membrane graft in place **(E)**. Postoperative 1 month image showing the limbal tissue and amniotic membrane graft dissolution gradually over 4 weeks **(F)**. Three months **(G)** and 1 year **(H)** after allogenic SLET, a stable well-epithelialized ocular surface with much less neovascularization, and faint residual deep stromal scarring is seen. The bottom row shows the 1-year timeline of the patient's right eye that underwent a modified sandwich allogenic SLET procedure and penetrating keratoplasty procedure simultaneously **(I–L)**. An image taken 2 months postoperatively showing intact limbal transplants and a well-placed amniotic membrane graft **(I)**; an image taken 2 months postoperatively showing that the sandwich graft, which included pieces of limbal tissue and two layers of amniotic membrane grafts, disintegrated gradually over 2 months **(J)**, 3 months **(K)**, and 1 year **(L)** after the procedure. A stable ocular surface with good epithelialization, mild neovascularization, and faint residual deep stromal scarring is seen. SLET, simple limbal epithelial transplantation; OCT, optical coherence tomography; LSCD, limbal stem cell deficiency.

A 35-year-old woman who had undergone LASIK in both eyes experienced bilateral total LSCD due to chemical burns. The presenting visual acuity was hand motion in her bilateral eyes. The slit lamp examination showed severely engorged, tortuous neovessel ingrowth onto the cornea with deep stromal scarring of the central cornea in both the eyes, descemetocele formation due to suspected flap melting in the right eye (Figure 1A), and thinning area impending perforation in her left eye (Figure 1B). Anterior segment optical coherence tomography (OCT) showed flap thinning related to suspicious melting flaps in both eyes (Figures 1C,D).

Due to severe bilateral LSCD, allogenic SLET from an HLA-matched living related donor was the first choice of treatment for her left eye. The patient's father was identified as an appropriate donor after a thorough preoperative evaluation, including ABO typing, HLA typing, and T and B lymphocyte cross-matching. Allogenic SLET was performed by transplanting small pieces of limbal tissue and arranging them circularly with fibrin glue on the cornea, placed by an amniotic membrane graft with fibrin glue following vascularized pannus removal. At the end of the procedure, the left eye was covered with a soft bandage contact lens. Tapering doses of oral corticosteroids were continued in the 2 weeks post-surgery with maintenance of topical 1% prednisolone acetate eye drops. The limbal transplants and amniotic membrane graft were placed in the first postoperative week (Figure 1E) and gradually disintegrated over 4 weeks (Figure 1F). Three months later, the cornea of the left eye was a stable, well-epithelialized ocular surface with much less neovascularization, and faint residual deep stromal scarring (Figure 1G) 1 year after the surgery (Figure 1H). The patient's unaided hand-motion visual acuity (VA) in the left eye improved to 6/20.

The modified allogenic SLET procedure and penetrating keratoplasty procedure were performed simultaneously owing to perforation and iris incarceration in her right eye. A modified sandwich SLET procedure was used in her right eye as opposed to the traditional procedure in which the secondary amniotic membrane is placed over fixed, concentrically arranged limbal transplants before the soft bandage contact lens coverage. The dose of oral corticosteroids was tapered within 2 weeks postoperatively, and maintenance treatment with topical 1% prednisolone acetate eye drops was continued, as was done in her left eye. Two weeks later, the sandwich graft, which included limbal tissue and two layers of amniotic membrane grafts, remained *in situ* (Figure 1I) and dissolved gradually over 2 months (Figure 1J). A completely epithelialized, stable corneal surface with faint neovascularization was observed 3 months (Figure 1K) and 1 year postoperatively (Figure 1L). Her unaided VA of her right eye improved from light perception to counting fingers.

Written informed consent was obtained from the patient for the publication of any potentially identifiable images or data included in this article.

## Discussion

Published information about allogenic SLET for the treatment of bilateral LSCD is limited (7, 13–16) to 21 eyes in two studies detailing living related allogeneic SLET for chronic bilateral LSCD (7, 14). Shanbhag et al.'s study (7) showed 16 eyes undergoing allogeneic SLET using living related donor tissue with statistically significant improvement in the stable corneal surface and overall visual acuity post-intervention. Prabhasawat et al.'s study (14) included five living related allogenic SLET eyes achieving a 60% clinical success rate. However, among these living related allogenic SLET eyes, 16 eyes received living related donor tissue without HLA or ABO matching, thus requiring a combination of systemic and topical immunosuppressants postoperatively. HLA or ABO matching or immunosuppressant treatment postoperatively has not been indicated in Prabhasawat et al.'s study. We report the first case of HLA or ABO matching living related allogenic SLET for permanent restoration of the cornea for bilateral LSCD treatment.

Compared with conventional keratoplasty, transplantation of an allogenic limbal graft has a higher risk of graft rejection because the limbal area is densely-vascularized with a high density of Langerhans and antigen-presenting cells (20, 21). Systemic immunosuppressive therapy has been validated to improve allogenic stem cell transplantation, such as lr-CLAL and KLAL, outcomes and plays a pivotal role in survival of allogenic graft (22). Preoperative screening and proper donor selection for lr-CLAL and KLAL minimizes the antigenic burden for the transplanted tissue and maximizes the success of the procedure by identifying the best living related donor match (23). We modified the preoperative selection protocol of lr-CLAL and KLAL in our patient and identified a potential donor from first-degree parents. The potential donor–recipient pair was first ABO (blood group) typed to find the ABO-compatibility, followed by HLA matching [class I: HLA-A, HLA-B; class II: HLA-DR (D-related), HLA-DQ (DC)] to identify the closest HLA-matched donor. Then, the recipient's panel reactive antibody level was identified to avoid mismatch because of the developed antibodies against a specific HLA antigen of the recipient from prior sensitization (e.g., blood transfusion, pregnancy, and previous transplant). Finally, the virtual crossmatch included B lymphocyte and T lymphocyte crossmatch testing.

## Conclusions

SLET is a new simple technique that uses the least amount of healthy limbal donor tissue and does not require a laboratory system for cultivating limbal stem cells. It is easily adaptable, and the use of systemic immunosuppression is not necessary if an autogenic graft is received. However, for the treatment of total bilateral LSCD, the use of allogenic SLET is inevitable because of the small quantity of residual healthy limbal stem cells of the patient. Our ABO-HLA-matched living-related allogenic SLET is easier than COMET and achieves stable corneal surfaces, decreases the rejection rate, and removes the need for systemic immunosuppressants. It also overcame the limitations of prolonged systemic immunosuppressant use in lr-CLAL and KLAL. Our case showed that systemic immunosuppressants are not warranted in for living-related ABO-HLA-matched allogenic SLET. It can avoid any contraindications or side effects of systemic immunosuppression and is a potential optimal surgical treatment for bilateral total LSCD, compared with other transplantation techniques.

## Data Availability Statement

The original contributions presented in the study are included in the article/supplementary material, further inquiries can be directed to the corresponding authors.

## Ethics Statement

Ethical review and approval was not required for the study on human participants in accordance with the local legislation and institutional requirements. The patients/participants provided their written informed consent to participate in this study.

## Author Contributions

The data were analyzed and the manuscript was drafted by Y-SC and R-LJ. The manuscript was revised by R-LJ and S-HT. The results were analyzed and conclusions were made by Y-SC, R-LJ, and S-HT. Data were collected and images were interpreted by T-YC and R-LJ. The final manuscript was read and approved by all authors.

## Conflict of Interest

The authors declare that the research was conducted in the absence of any commercial or financial relationships that could be construed as a potential conflict of interest.

## Publisher's Note

All claims expressed in this article are solely those of the authors and do not necessarily represent those of their affiliated organizations, or those of the publisher, the editors and the reviewers. Any product that may be evaluated in this article, or claim that may be made by its manufacturer, is not guaranteed or endorsed by the publisher.
